# Rapid generation of HCoV-229E and HCoV-OC43 reporter viruses and replicons for antiviral research

**DOI:** 10.3389/fcimb.2025.1614369

**Published:** 2025-07-22

**Authors:** Yuyuan Zhang, Jiannan Chen, Hui Feng, Lulu Yang, Liyuan Hu, Yang Gao, Ziqiao Wang, Fei Feng, Jincun Zhao, Ping Zhang, Rong Zhang

**Affiliations:** ^1^ Key Laboratory of Medical Molecular Virology (MOE/NHC/CAMS), Shanghai Institute of Infectious Disease and Biosecurity, Shanghai Frontiers Science Center of Pathogenic Microorganisms and Infection, School of Basic Medical Sciences, Shanghai Medical College, Fudan University, Shanghai, China; ^2^ Department of Immunology and Microbiology, Zhongshan School of Medicine, Sun Yat-sen University, Guangzhou, China; ^3^ State Key Laboratory of Respiratory Disease, National Clinical Research Center for Respiratory Disease, Guangzhou Institute of Respiratory Health, the First Affiliated Hospital of Guangzhou Medical University, Guangzhou, China

**Keywords:** human coronavirus, reverse genetics system, TAR cloning, reporter virus, replicon, antiviral

## Abstract

**Introduction:**

The large size of coronavirus genome, along with the instability of certain genomic sequences, makes the construction of reverse genetics for coronaviruses particularly challenging. The rapid development and application of reverse genetics systems for coronaviruses require further exploration.

**Methods:**

Using transformation-associated recombination (TAR) cloning in yeast and the *in vitro* CRISPR-Cas9 system, reverse genetics systems of two mild coronaviruses HCoV-OC43 and HCoV-229E were rapidly established. Antiviral assays, high-content imaging, and NanoLuc luciferase assays were used to characterize reporter viruses and replicon systems.

**Results:**

We rapidly assembled infectious clones for two mild coronaviruses, HCoV-OC43 and HCoV-229E, using transformation-associated recombination (TAR) cloning in yeast. The infected clones could stably express the mGreenLantern reporter gene. We further generated T7 promoter-driven RNA replicon of HCoV-229E and CMV promoter-driven DNA replicon of HCoV-OC43, with the readout of NanoLuc luciferase activity. The effectiveness of these tools for antiviral study was evaluated using the broad-spectrum RNA-dependent RNA polymerase inhibitor remdesivir, exhibiting high sensitivity, efficiency, and convenience.

**Discussion:**

The application of yeast-based TAR cloning significantly facilitates the rapid assembly of large viral genome, and the establishment of HCoV-OC43 and HCoV-229E reverse genetics systems provides valuable platforms for studying the biology and developing antivirals against coronaviruses.

## 1 Introduction

Coronaviruses are enveloped, single-stranded positive-sense RNA viruses with genome sizes ranging from approximately 26 to 32 kilobases, making them one of the largest RNA virus genomes (Woo et al., 2010). In humans, mild diseases include some seasonal colds, primarily caused by HCoV-OC43 (OC43) and HCoV-229E (229E) (Walsh et al., 2013), while highly pathogenic strains, such as SARS-CoV, MERS-CoV, and SARS-CoV-2, can lead to more severe viral pneumonia (Chan-Yeung and Xu, 2003; Zhou et al., 2020). In animals, coronaviruses can cause diseases such as hepatitis and encephalomyelitis in mice (MHV) (Dick et al., 1956), and diarrhea in pigs (PEDV) (Jung et al., 2020). Among human coronaviruses, three (SARS-CoV, MERS-CoV, and SARS-CoV-2) have caused outbreaks of severe epidemics in the past two decades, especially the global COVID-19 pandemic, which emerged in Wuhan, Hubei Province, China, at the end of 2019. This pandemic has had a significant impact on public health and healthcare systems (Nicola et al., 2020). The rapid emergence potential of coronaviruses serves as a reminder that greater attention should be paid to the molecular biology and pathogenesis in order to develop countermeasures for the prevention and control.

The development of reverse genetics for coronaviruses has made it possible to manipulate the genomes at the cDNA level, greatly facilitating the research on viral genomic structures, protein functions, virulence factors, and pathogenicity (Almazán et al., 2014). By introducing reporter genes, such as fluorescent proteins, into the viral genomes using reverse genetics, their expression can serve as a reliable readout to monitor viral infection in cell culture models, replacing labor-intensive viral detection methods (Hotard et al., 2012; Kanai et al., 2017; Chiem et al., 2021). Additionally, replicon systems have been developed for some coronaviruses. The incorporation of reporter genes (e.g., fluorescent proteins or luciferase) and antibiotic-resistance markers allows for rapid generation of stable cell lines and straightforward quantification of viral replication (Hertzig et al., 2004; Gutiérrez-Álvarez et al., 2021; He et al., 2021). Thus, the reporter viruses and replicon systems generated from reverse genetics provide useful tools for rapid evaluation of antiviral compounds and detailed studies of virus–host interactions.

Given the large size of coronavirus genomes, a major obstacle in constructing reverse genetics system is assembling the full-length cDNA clone. Initially, researchers took advantage of the high homologous RNA recombination rate of coronaviruses and developed the first reverse genetics system for coronaviruses based on targeted RNA recombination strategy (Koetzner et al., 1992). However, this method relied on viral replication and propagation, limiting its use in studying lethal mutations and controlling the entire coronavirus genome. Over the next two decades, various strategies for constructing infectious cDNA clones were developed, including bacterial artificial chromosomes (BAC), *in vitro* ligation, vaccinia virus vector, bacterial/yeast artificial chromosomes (BAC/YAC), and circular polymerase extension reaction (CPER) (Rice et al., 1989; Yount et al., 2000; Casais et al., 2001; Edmonds et al., 2013). These reverse genetics systems vary in complexity for manipulating genomes.

229E and OC43 are both human endemic coronaviruses, belonging to the *Alphacoronavirus* and *Betacoronavirus* genera, respectively, and can cause mild upper respiratory diseases. 229E originates from bats and shares a common ancestor with bat coronaviruses (GhanaGrp1 Bt CoV) (Pfefferle et al., 2009), while OC43 is believed to have originated in rodents, rather than bats, and may share a common ancestor with bovine coronavirus (BCoV) (Forni et al., 2017). It is believed to have spilled over to humans more than 200 years ago through a cross-species transmission event (Vijgen et al., 2005). In addition to causing respiratory infections, OC43 is also thought to cause neurological symptoms (Corman et al., 2018). Given the similarities of genomic structures and protein functions among coronavirus family members, these two types of mild coronaviruses represent valuable and safe alternative models for studying highly pathogenic coronaviruses which require biosafety level 3 (BSL-3) facility (Chiu et al., 2023).

Several methods for constructing infectious clones of OC43 and 229E have been developed. The reverse genetics system for 229E was generated based on a vaccinia virus-based approach (Thiel et al., 2001), while OC43 was developed using *in vitro* ligation strategy and the BAC system (Shen et al., 2016; Diefenbacher et al., 2024). Using these reverse genetics platforms, several reporter viruses carrying fluorescent proteins or luciferases have been rescued and applied to antiviral compound screening, with the potential for high-throughput screening experiments.

The successful application of yeast in synthetic biology has made it possible to assemble coronavirus genomes in one step (Gibson et al., 2008). Currently, yeast homologous recombination is the most efficient method for assembling large DNA fragments, and yeast is less sensitive to toxic viral sequences. Cytomegalovirus (CMV), Herpes simplex virus type 1 (HSV-1) (Oldfield et al., 2017; Vashee et al., 2017), and cDNA clones of large viral genomes, including SARS-CoV-2 (Thi Nhu Thao et al., 2020), have been successfully assembled in yeast.

In this study, we utilized the efficient DNA recombination capability of *Saccharomyces cerevisiae* to rapidly construct reverse genetics systems for both OC43 and 229E viruses. Recombinant viruses expressing the mGreenLantern (mGreen) reporter gene were successfully rescued, and their application in image-based antiviral assays was validated, enabling high-throughput screening for broad-spectrum antiviral drugs. Furthermore, we constructed two different types of replicon systems for OC43 and 229E. These replicons were used to assess the antiviral activity of remdesivir that can inhibit the polymerase activity. Our work provides useful platforms for studying the functions of viral and host factors and screening the drugs against coronavirus infections.

## 2 Materials and methods

### 2.1 Cells and viruses

HRT-18 (ATCC#CCL-24), HEK 293T (ATCC#CRL-3216), HeLa (ATCC#CCL-2), BHK-21, and Huh7 cells were cultured in Dulbecco’s modified Eagle’s medium supplemented with 10% fetal bovine serum (FBS), 10 mM HEPES, 1 mM sodium pyruvate, 1× non-essential amino acids, and 100 U/ml of penicillin-streptomycin. Wild-type HCoV-OC43 (VR-1558) was propagated in HRT-18 cells, and wild-type HCoV-229E (VR-740) was propagated in Huh7 cells.

### 2.2 Transformation-associated recombination assembly of viral fragments in yeast

The pBAC TAR vector was constructed by modifying the pYES1L vector (Thermo), which contains a yeast artificial chromosome (YAC) and a bacterial artificial chromosome (BAC). For transformation-associated recombination (TAR), a fragment containing the Human cytomegalovirus (CMV) promoter, the 5′ UTR of the viral genome, an EcoRI restriction enzyme site, the 3′ UTR of viral genome, 37-nt poly (A) sequences, hepatitis delta virus ribozyme (HDVr) sequences, and simian virus 40 (SV40) polyadenylation signal sequences was generated by molecular cloning and inserted into the linearized pYES1L vector.

The pBAC plasmid was linearized with EcoRI restriction enzyme. The fragment containing the mGreen reporter gene was obtained by PCR, while the remaining fragments were amplified from viral RNAs. The viral RNAs were extracted from the supernatant of cells infected with wild-type HCoV-229E or HCoV-OC43 virus. Transformation-associated recombination was performed by PEG/LiAc approach (Gietz and Schiestl, 2007). A total of nine DNA fragments were co-transformed with the linearized pBAC vector into competent yeast cells. The transformed cells were then cultured on CSM-Trp selection plates at 30°C for two days. The positive yeast colonies were screened and electroporated into *E. coli* for plasmid amplification. Plasmids were prepared for virus rescue.

### 2.3 Rescue of recombinant HCoV-229E and HCoV- OC43 reporter viruses

The reporter viruses were rescued from pBAC-229E-mGreen and pBAC-OC43-mGreen infectious cDNA clones. BHK-21 cells were transfected with infectious clones using Fugene HD transfection reagent (Promega) in 6-well plate. After incubation for 8 h at 37°C in a humidified 5% CO2 incubator, the transfected cells were maintained in fresh DMEM supplemented with 2% FBS for 4 days. The supernatants were subsequently collected and amplified on Huh7 cells for 229E-mGreen or on HRT-18 cells for OC43-mGreen. Infected cells were observed daily for mGreen expression and presence of virus-induced cytopathic effects (CPE). Four days post-infection, the supernatants were collected, labeled as passage 1 (P1), and stored at -80°C.

### 2.4 Indirect immunofluorescence assay

Cells infected with viruses were fixed with 2% PFA for 10 min at room temperature, permeabilized with 0.2% Triton X‐100 for 15 min, and then incubated overnight at 4°C with indicated house-made mouse anti-229E or anti-OC43 nucleocapsid (N) antibody (1:1000). After three washes, cells were incubated with the secondary goat anti-mouse IgG (H+L) conjugated with Alexa Fluor 555 (Thermo), followed by staining with 4’,6-diamidino-2-phenylindole (DAPI). Representative images were acquired using an EVOS M5000 imaging system (Thermo).

### 2.5 Virus growth kinetics and titration

Growth kinetics for 229E-WT or 229E-mGreen were performed on Huh7 cells at a multiplicity of infection (MOI) of 0.01, while OC43-WT or OC43-mGreen were performed on HRT-18 cells at an MOI of 0.005. Confluent monolayers of cells in 6-well plates were infected with individual viruses at corresponding MOI. After 2 h of adsorption at 37°C, the inoculum was removed, and the cells were washed three times with PBS, then maintained in 2% FBS culture media, and viral supernatants were collected at 3, 6, 9, 12, 24, 36, 48, 60 72, 84, and 96 h post-infection for titration by focus-forming assay.

For virus titration, cells were inoculated with 10-fold serially diluted virus supernatants for 2 h and overlaid with 1% methylcellulose in DMEM medium containing 2% FBS. For foci visualization, cells were fixed with 2% PFA after 48 h at 37°C, then subjected to immunostaining with house-made mouse anti-229E or anti-OC43 nucleocapsid antibody (1:2000) and secondary goat anti-mouse IgG HRP (Sigma), followed by staining with KPL TrueBlue Substrate (SeraCare) until foci are fully formed. The foci were counted and the titers were calculated.

### 2.6 Focus-forming assay

The assays for 229E-WT or 229E-mGreen were performed on Huh7 cells at a multiplicity of infection (MOI) of 0.02 for 24h, while OC43-WT or OC43-mGreen were performed on HRT-18 cells at an MOI of 0.01 for 72h. Confluent monolayers of Huh7 and HRT-18 cells were inoculated with indicated viruses for 2h and overlaid with 1% methylcellulose in DMEM medium containing 2% FBS. For immunostaining of nucleocapsid (N) protein, the cells were fixed for 30 min with 2% PFA, then subjected to immunostaining with house-made mouse anti-229E or anti-OC43 nucleocapsid antibody (1:2000) and secondary HRP-conjugated goat anti-mouse IgG (Sigma), followed by staining with KPL TrueBlue Substrate (SeraCare). The mGreen-positive or nucleocapsid-positive foci were captured using the CTL ImmunoSpot software.

### 2.7 RNA extraction and RT-PCR

To analyze the stability of the mGreen reporter gene during virus passaging, viral RNA from collected supernatant was extracted with the TIANamp Virus RNA kit (TIANGEN#DP315). For reverse transcription-PCR (RT-PCR), two sets of primer pairs flanking the reporter gene were used: one pair for OC43-mGreen (5′-GGGTAAACTACTTATTAGAGATAC-3′)/(5′-GACAAGCCTTACGGAAATGAAAAC-3′) and the other pair for 229E-mGreen (5′-CGACGTTGAAAAGATCCACATAC-3′)/(5′-CGTTAGTTGAGAGATAAGAAACAATT-3′). The PCR products were subjected to electrophoresis on a 1% agarose gel.

### 2.8 Image-based antiviral assay and cytotoxicity assay

Antiviral assay for OC43-mGreen were performed on HRT-18 cells at an MOI of 0.005 for 96 h, while 229E-mGreen were performed on Huh7 cells at an MOI of 0.02 for 36 h. Remdesivir (Selleck) was solubilized in DMSO to yield the solution of 20 mM stock. The 3-fold serially diluted remdesivir was added to confluent monolayers of cells in 96-well plates. After 1 h of incubation at 37°C, cells were infected with individual viruses at certain MOI. Cells were incubated at 37°C and 5% CO2 for indicated timepoints, then fixed with 2% PFA for 10 min, permeabilized with 0.2% Triton X‐100 for 15 min, followed by staining with DAPI for 5 min at room temperature. Images were collected using an Operetta High Content Imaging System (PerkinElmer), and processed using the PerkinElmer Harmony high‐content analysis software v4.9.

The cytotoxicity was determined by using the CellTiter-Lum Steady II assay according to the manufacturer’s instructions (Beyotime). Briefly, HRT-18 or Huh7 cells in 96-well plates were incubated with 3-fold serially diluted remdesivir, equivalent concentration of DMSO was added to cells as negative control. Viability of the cells after 24 h of treatment was determined by measuring the luminescence on a Spark Microplate Reader (Tecan) after the addition of the CellTiter-luminescent reagent (Beyotime).

### 2.9 Construction of 229E and OC43 replicons

The guide RNAs for *in vitro* CRISPR/Cas9 nuclease cleavage were designed according to the instructions from the EnGen sgRNA website (https://sgrna.neb.com/#!/sgrna). Guide RNAs targeting the pBAC vector and viral genome coding sequences were individually transcribed *in vitro* using the EnGen sgRNA Synthesis Kit (NEB). The pBAC-OC43-mGreen or pBAC-229E-mGreen plasmids were digested with Cas9 nuclease and the corresponding guide RNAs at 37°C for 1 h. The digested products were then purified and ligated with PCR-amplified viral genomic fragments bearing homologous arms.

To construct the OC43 replicon bearing the mutations (D760N/D761N) in the NSP12 protein, pBAC-OC43-Replicon plasmid was digested using Cas9 nuclease and the corresponding guide RNAs, as described above. The fragment containing NSP12 D760N/D761N mutations was obtained by PCR and subsequently inserted into the linearized pBAC-OC43-Replicon through homologous recombination.

### 2.10 Replicon transfection and NanoLuc luciferase luminescence quantification

The replicon RNA was transcribed by the mMESSAGE mMACHINE T7 Transcription Kit (Thermo) according to the manufacturer’s instructions with some modifications. Briefly, 25 µL of T7 transcription reaction mixture containing 1 µg of linearized replicon DNA was incubated at 37°C for 2.5 h. After incubation, 1.25 µL TURBO DNase was added, and the reaction was incubated at 37°C for 15 minutes to digest the template DNA. The resulting RNA was purified using LiCl precipitation solution.

For the electroporation with Neon NxT Electroporation System (Thermo). HeLa cells grown to 80% confluence in T25 flask were trypsinized and washed twice with PBS. The washed cells (1 × 10^5^ cells) were mixed with 500 ng of replicon RNA in 10 μL of Resuspension Buffer R. Electroporation parameters were set as follows: voltage = 1005 V, pulse width = 30 ms, and pulse number = 3. After electroporation, the cells were seeded into 96-well plate. At various time points post-transfection, the cells were lysed with Nano-Glo^®^ Luciferase Assay System (Promega). The luminescence signal was detected by a Spark Microplate Reader (Tecan).

The transfection of OC43 replicon DNA was performed according to the protocol of EZ Trans transfection reagent (Life-ilab). Briefly, HEK293T cells grown to 80% confluence were transfected with 50 ng of OC43 replicon plasmid with 0.2 μL EZ Trans Transfection Reagent per well in 96-well plate. At indicated time points post-transfection, cells were lysed with Nano-Glo^®^ Luciferase Assay buffer (Promega), and the luminescence signal was detected using a Spark Microplate Reader (Tecan).

### 2.11 Statistical analysis

Data are provided as the mean ± standard deviation. Statistical significance was assigned when p values were <0.05 using Prism Version 9 (GraphPad). An ANOVA, unpaired Student’s t test was used to determine the statistical significance.

## 3 Results

### 3.1 Schematic of the assembly of HCoV-OC43 and HCoV-229E genomes bearing fluorescent protein reporter genes

**Figure 1:**
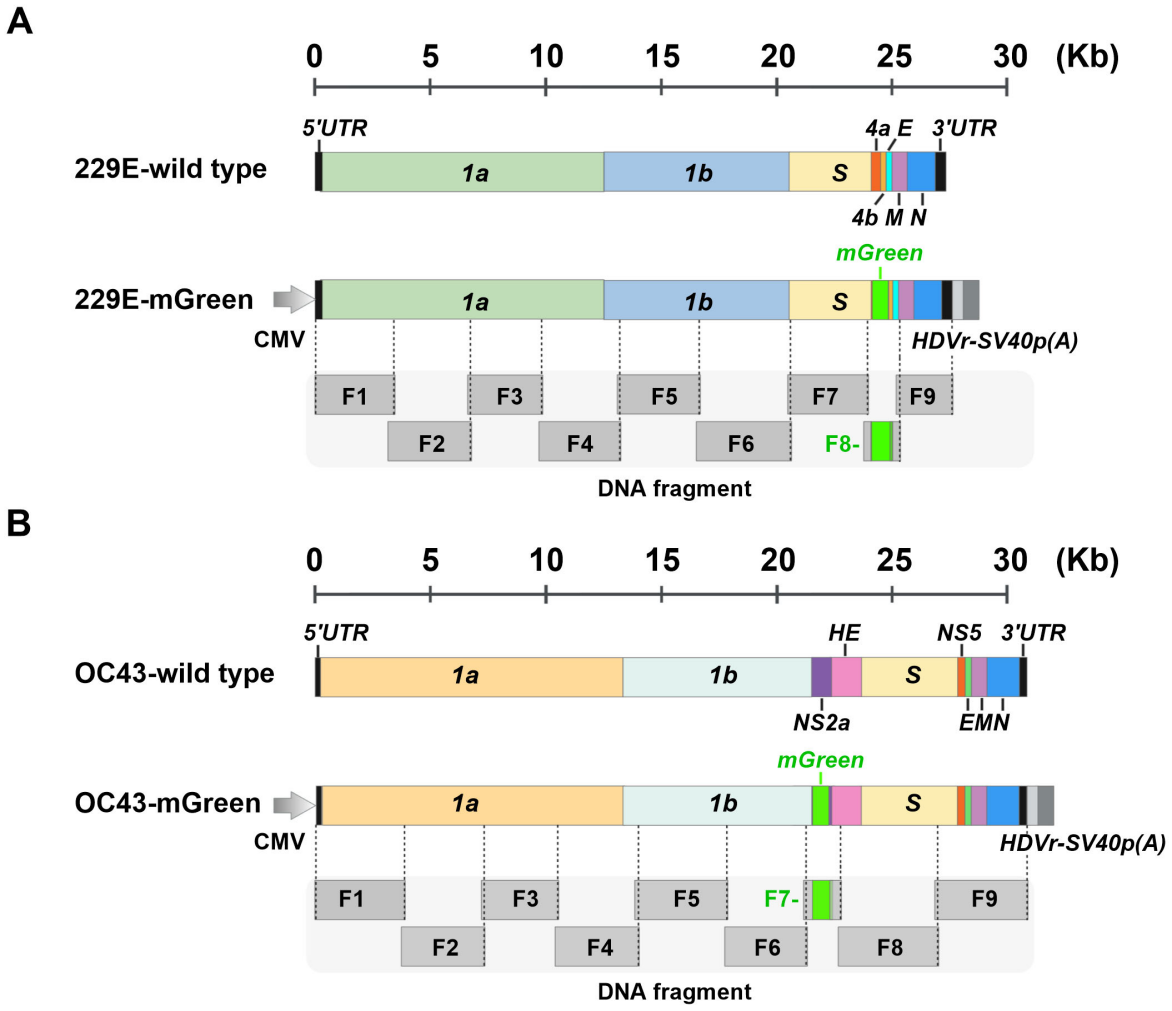
Schematic representation of the construction of full-length infectious clones of 229E-mGreen and OC43-mGreen reporter viruses. **(A)** Schematic of wild-type 229E and chimeric 229E-Green genomes. The open reading frames (ORFs) of the nonstructural, structural, and accessory genes are indicated. The mGreenLantern gene (mGreen) was inserted to replace the ns4a gene. **(B)** Schematic of wild-type OC43 and chimeric OC43-mGreen genomes. The ORFs of nonstructural, structural, and accessory genes are indicated. The mGreen gene was inserted upstream of the HE gene to replace ns2a. The N-terminal 18 amino acids of the ns2a were maintained. Both 229E and OC43 genomes are divided into nine fragments (F1-F9), with approximately 130 bp of overlap between adjacent fragments.

To explore the versatility of yeast TAR cloning for coronavirus reverse genetics system, we designed a strategy to assemble the full-length genomes of HCoV-229E and HCoV-OC43, representing the *Alphacoronavirus* and *Betacoronavirus* genera, respectively (**Figure 1**). Reporter viruses expressing fluorescent protein mGreenLantern (thereafter mGreen) were developed following the strategies described previously (Cervantes-Barragan et al., 2010; Shen et al., 2016). For HCoV-229E, the open reading frame of the accessory protein ns4a was replaced with the mGreen gene. For HCoV-OC43, the mGreen gene was inserted to replace the accessory ns2a, except for the first 18 codons due to the proximity of the ns2a translation initiation site to the transcriptional regulatory sequences (TRS). The full-length genomes of both viruses were divided into nine fragments, which share approximately 130 nucleotides between adjacent fragments. To facilitate gene transcription and termination, a cytomegalovirus (CMV) promoter and a cassette containing the hepatitis D virus ribozyme (HDVr) and SV40 polyadenylation signal sequences were added upstream of the 5′UTR and downstream of the 3’ UTR of genome, respectively.

### 3.2 Rescue and characterization of recombinant HCoV-OC43 and HCoV-229E reporter viruses

**Figure 2:**
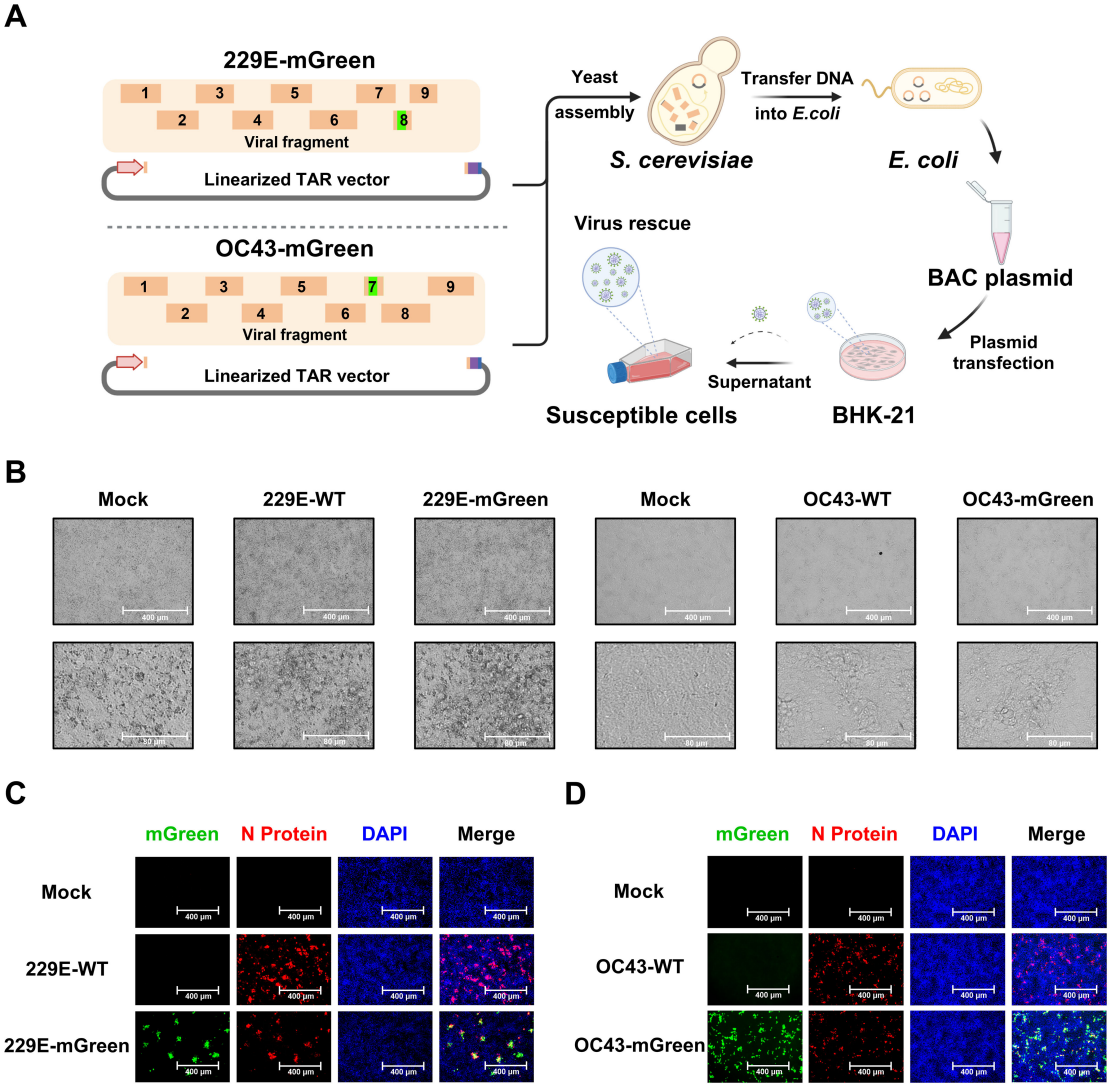
Recovery and characterization of 229E-mGreen and OC43-mGreen reporter viruses. **(A)** Workflow of TAR cloning and reporter virus rescue. The DNA fragments with overlapping junctions were assembled through homologous recombination in yeast, and full-length cDNA clones was extracted from yeast and electroporated into *E. coli* competent cells. Virus rescue was initiated by transfecting BHK-21 cells with purified plasmids, followed by amplification in susceptible cells. **(B)** Cytopathic effects (CPE) of 229E-mGreen and OC43-mGreen reporter viruses in Huh7 at 24 h and HRT-18 cells at 72 h, respectively. The parental wild-type viruses were used as a control. Scale bar, 400 μm (top) or 80 μm (bottom). **(C)** Huh7 cells were infected with 229E-WT and 229E-mGreen for immunofluorescence staining of nucleocapsid (N) protein (MOI 0.02, 24 h). **(D)** HRT-18 cells were infected with OC43-WT and OC43-mGreen for immunofluorescence staining of nucleocapsid (N) protein (MOI 0.01, 72 h). The images were captured using a fluorescence microscope. Scale bar, 400 μm.

To assemble the full-length viral genomes (**Figure 2A**), viral fragments with overlapping junctions were delivered into yeast for homologous recombination. The resulting full-length cDNA clones were extracted and electroporated into *E. coli* cells for amplification. To rescue the recombinant reporter viruses HCoV-OC43 (OC43-mGreen) and HCoV-229E (229E-mGreen) from cDNA driven by the CMV promoter, the assembled infectious clones were transfected into BHK-21 cells. After 96 h, the culture supernatants were harvested to propagate in susceptible cells. Typical cytopathic effects (CPE) were observed in infected cells for both reporter viruses (**Figure 2B**), similar to those of their parental viruses. Meanwhile, strong expression of the mGreen protein was observed in infected cells under fluorescence microscope (**Figures 2C, D**).

**Figure 3:**
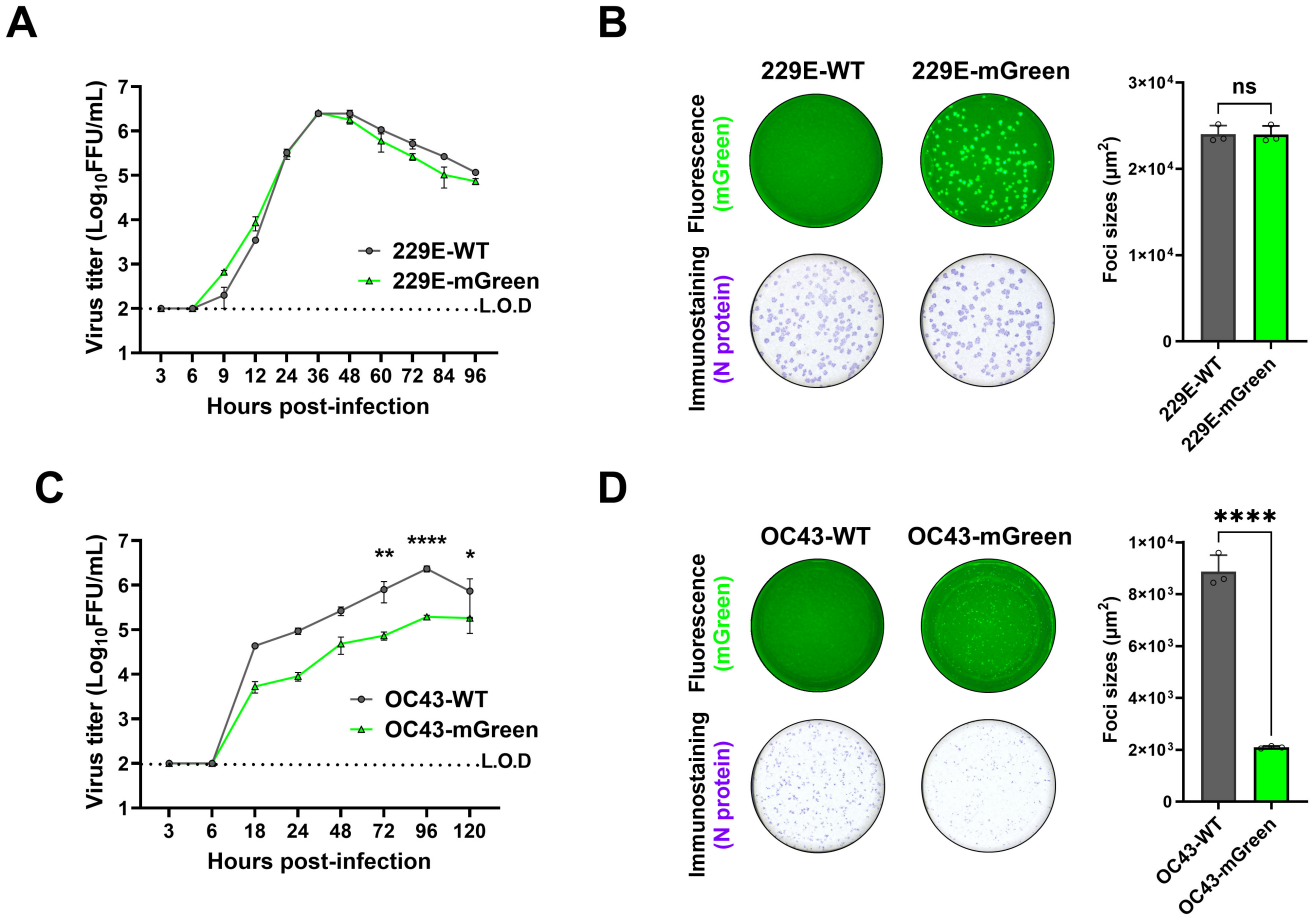
Growth property of recovered reporter viruses. **(A)** Growth kinetics of 229E reporter virus. Huh7 cells were infected with 229E-WT and 229E-mGreen (MOI 0.01). Supernatants were collected at the indicated time points, and viral titers were determined in huh7 cells by focus forming assay. **(B)** Focus-forming morphology of 229E reporter virus. Huh7 cells were infected with 229E-WT and 229E-mGreen (MOI 0.02, 24 h). The mGreen-positive fluorescence (top) and immunostaining of nucleocapsid (bottom) images were captured and the areas (square micrometer) of foci were quantified using a CTL ImmunoSpot analyzer. The mean sizes of >100 foci per virus with positive standard deviations was shown. **(C)** Growth kinetics of OC43 reporter virus. HRT-18 cells were infected with OC43-WT and OC43-mGreen (MOI 0.005). Supernatants were collected at the indicated time points, and viral titers were determined in HRT-18 cells by focus forming assay. **(D)** Focus-forming morphology of OC43 reporter virus. HRT-18 cells were infected with OC43-WT and OC43-mGreen (MOI 0.01, 72 h). The mGreen fluorescence (top) and immunostaining of nucleocapsid (bottom) images were captured and the areas (square micrometer) of foci were quantified using a CTL ImmunoSpot analyzer. The mean sizes of >100 foci per virus with positive standard deviations was shown. * : P < 0.05; ** : P < 0.01; *** : P < 0.001, ns : not statistically significant.

The growth characteristics of the recovered viruses were further evaluated. Multi-step growth kinetics indicated that replication of the 229E-mGreen virus was comparable to its parental virus, reaching a peak titer of 2.6 × 10^6^ focus-forming units (FFU)/mL at 36 h post-infection (**Figure 3A**). There was no significant difference in focus-forming morphology between the rescued 229E-mGreen and its parental virus 229E-WT (**Figure 3B**). In contrast, the OC43-mGreen virus replicated somewhat slower than its parental virus, with a peak titer of 1.9 × 10^5^ FFU/mL at 96 h post-infection (**Figure 3C**). Moreover, the recovered OC43-mGreen exhibited a smaller focus size (**Figure 3D**), suggesting that the insertion of reporter gene in place of ns2a protein adversely affects virus replication.

These results demonstrate that yeast-based TAR cloning is an efficient strategy for assembling the genomes of HCoV-OC43 and HCoV-229E, and that the recombinant mGreen-reporter viruses rescued provide valuable tools for the functional study of coronavirus infection.

### 3.3 Stability of the reporter gene during serial passaging

**Figure 4:**
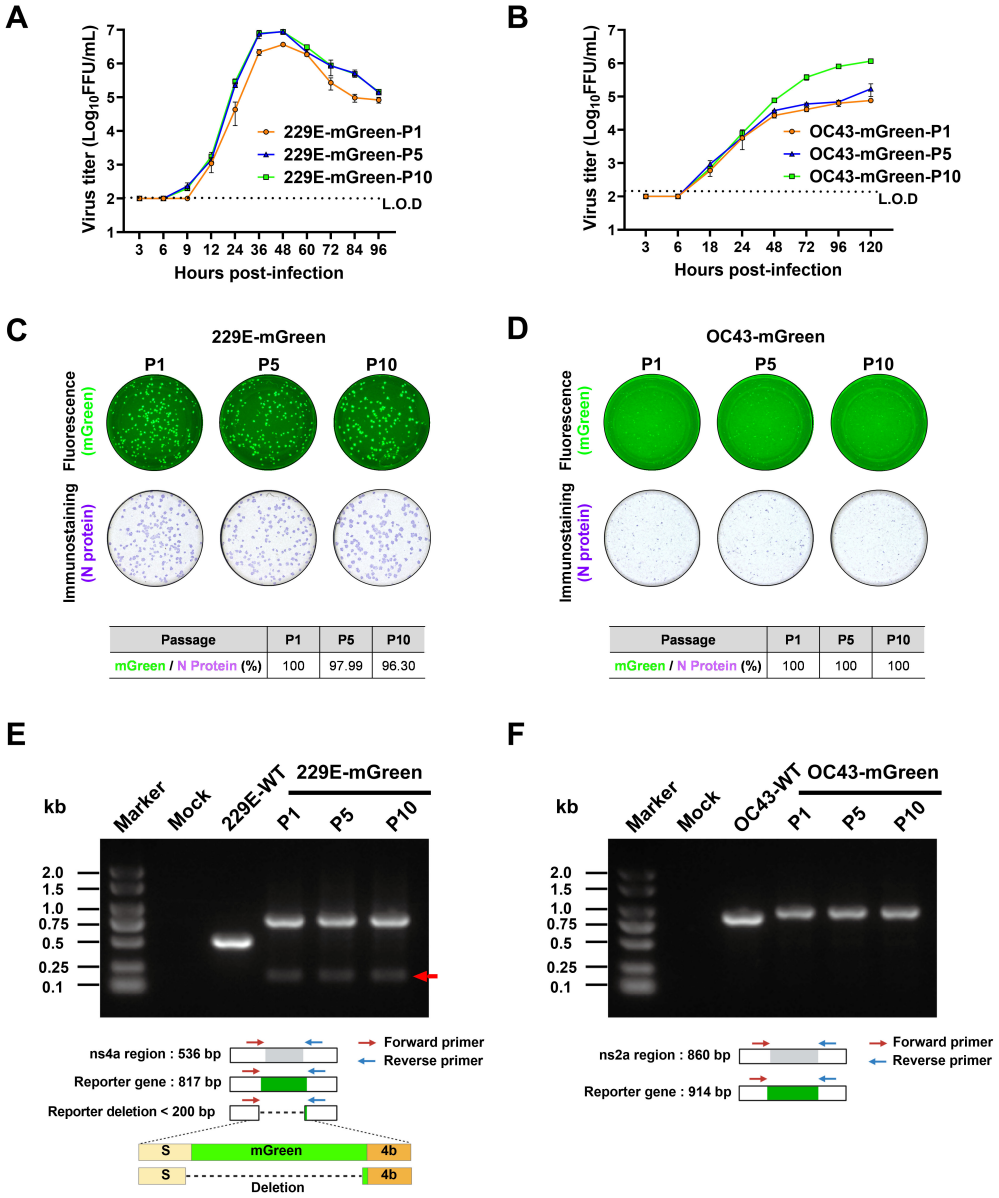
Stability analysis of the mGreen reporter gene during serial passaging. **(A, B)** Growth kinetics of the indicated passages (P1, P5, P10) of reporter viruses. Huh7 cells were infected with 229E-mGreen (P1, P5, P10) at a MOI 0.01 **(A)**. HRT-18 cells were infected with OC43-mGreen (P1, P5, P10) at a MOI 0.005 **(B)**. Supernatants were collected at the indicated time points and viral titers were determined in respective cells by focus-forming assay. **(C, D)** P1, P5, and P10 stocks of 229E-mGreen (MOI 0.02, 24 h) **(C)** and OC43-mGreen (MOI 0.01, 72 h) **(D)** were used to infect Huh7 and HRT-18 cells, respectively. The foci formed upon infection with the indicated passages (P1, P5, P10) of reporter viruses were analyzed for the expression of mGreen (top) and nucleocapsid protein (bottom). Images were captured using a CTL ImmunoSpot analyzer, and the percentage of mGreen-positive foci in nucleocapsid-positive foci were calculated. **(E, F)** Analysis of the genetic stability of the inserted mGreen gene after 10 passages in susceptible cells. Viral RNA was extracted from culture supernatants of P1, P5 and P10, and RT-PCR was performed using primer pairs flanking the reporter gene region. The amplified products were resolved by 1% agarose gel electrophoresis.

Next, the genetic stability of 229E-mGreen and OC43-mGreen was determined. Both reporter viruses were serially passaged 10 times in susceptible cells. It appears that increased replicative fitness *in vitro* was observed for both reporter viruses (**Figures 4A, B**).

The expression of the reporter gene mGreen was assessed by focus-forming assay and RT-PCR. For P1, P5, and P10 of these two viruses, there was no obvious loss of mGreen fluorescent signal in infected cells (**Figures 4C, D**). To evaluate the genetic stability of the mGreen expression cassette in the recombinant viruses during serial passaging, the regions encompassing the mGreen insertion in 229E-mGreen (**Figure 4E**) or OC43-mGreen (**Figure 4F**) were amplified by RT-PCR. PCR products of the expected size encompassing the reporter gene in the genomes of both recombinant viruses were detected. For the 229E-mGreen reporter virus, the amplicon size was 0.8 kb, while the parental virus produced a 0.5 kb amplicon (**Figure 4E**). Meanwhile, a faint band of less than 200 bp was detected in P1, P5, and P10 of 229E-mGreen recombinant virus. Sanger sequencing of the PCR product of P10 confirmed the presence of a deletion in the viral genome, with a portion of the carboxy-terminal spike-coding region (nt3496-3522) and the majority of mGreen-coding region (nt1-726) being deleted (**Figure 4E**). However, it appears that the deletion of mGreen reporter gene occurred slowly and remained relatively stable over 10 passages. As for the OC43-mGreen recombinant virus and its parental strain, the amplicon sizes were approximately 0.9 kb and 0.8 kb, respectively (**Figure 4F**), and no additional small band was observed even for P10 virus, suggesting that the insertion of mGreen in place of ns2a remains stable.

### 3.4 Evaluation of the antivirals using reporter viruses

**Figure 5:**
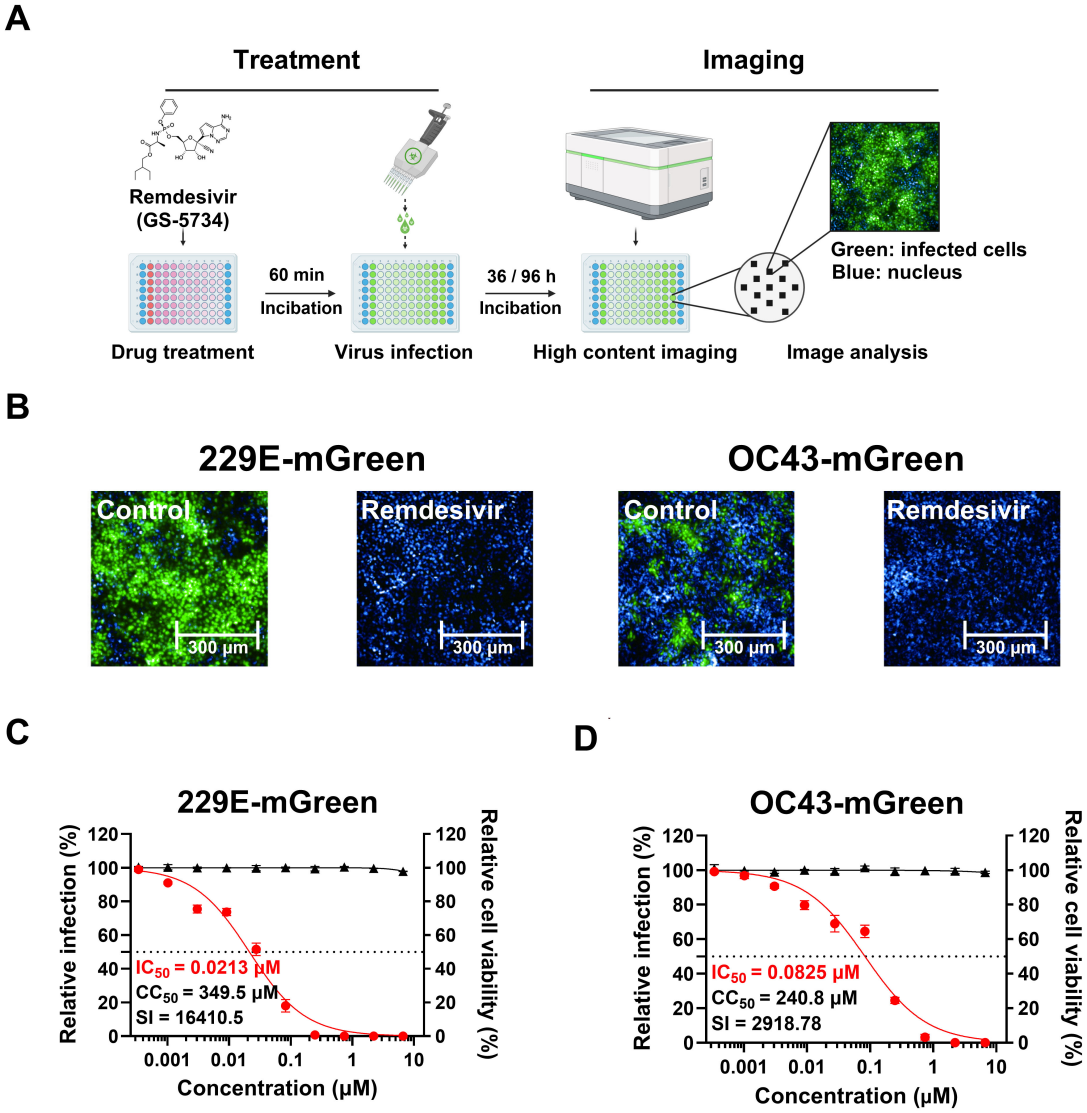
Validation of an image-based antiviral assay using reporter viruses. **(A)** The procedure of antiviral assay with mGreen reporter viruses. Cells in 96-well plates were pre-treated with three-fold dilutions of remdesivir for 60 minutes, followed by infection in the presence of the compound. The infection duration was optimized for detecting fluorescent protein expression (229E-mGreen, MOI 0.02, 36 h; OC43-mGreen, MOI 0.005, 96 h). After fixation, cells were stained with DAPI, and images were captured using the Operetta CLS High-Content Analysis System. Fluorescent images from each well were analyzed to calculate the percentage of infected cells. **(B)** Representative images of Huh7 or HRT-18 cells infected with 229E-mGreen or OC43-mGreen, respectively. Cells were pre-treated with 2 μM remdesivir or DMSO prior to infection. **(C, D)** Antiviral effect of remdesivir on 229E-mGreen **(C)** or OC43-mGreen **(D)**. The cells pre-treated with remdesivir were infected with reporter viruses, and mGreen-positive cells were quantified (229E-mGreen 36 h; OC43-mGreen, 96 h). Cell viability following treatment with remdesivir was determined. The IC_50_ and CC_50_ values were calculated.

To demonstrate the feasibility of our reporter viruses for screening and characterizing antiviral drugs, we established an antiviral assay based on fluorescent reporter in combination with a high-content imaging system, using remdesivir as a reference compound, which has been described and shown to efficiently inhibit various coronaviruses (Kokic et al., 2021). Imaging analysis based on the expression of fluorescent protein mGreen in the reporter viruses (**Figure 5A**) was performed.

We tested the inhibitory effect of remdesivir on 229E-mGreen virus in Huh7 cells and OC43-mGreen virus in HRT-18 cells. Huh7 cells were treated with serial dilutions of remdesivir (up to 20 μM) for 1 hour, followed by infection with 229E-mGreen reporter virus at an MOI of 0.02 for 36 h. Similarly, after remdesivir treatment, HRT-18 cells were infected with OC43-mGreen reporter virus at an MOI of 0.005 for 96 h. As expected, remdesivir treatment completely inhibited the infection by OC43-mGreen and 229E-mGreen (**Figure 5B**), with IC_50_ values of 0.0825 μM and 0.0213 μM, respectively (**Figures 5C, D**). No significant cytotoxicity was observed in either cell lines (**Figures 5C, D**). These results indicate that antiviral assay using reporter viruses in combination with a high-content imaging system is a direct and sensitive method suitable for screening of potential anti-coronavirus compounds.

### 3.5 Construction and antiviral evaluation of replicon system

**Figure 6:**
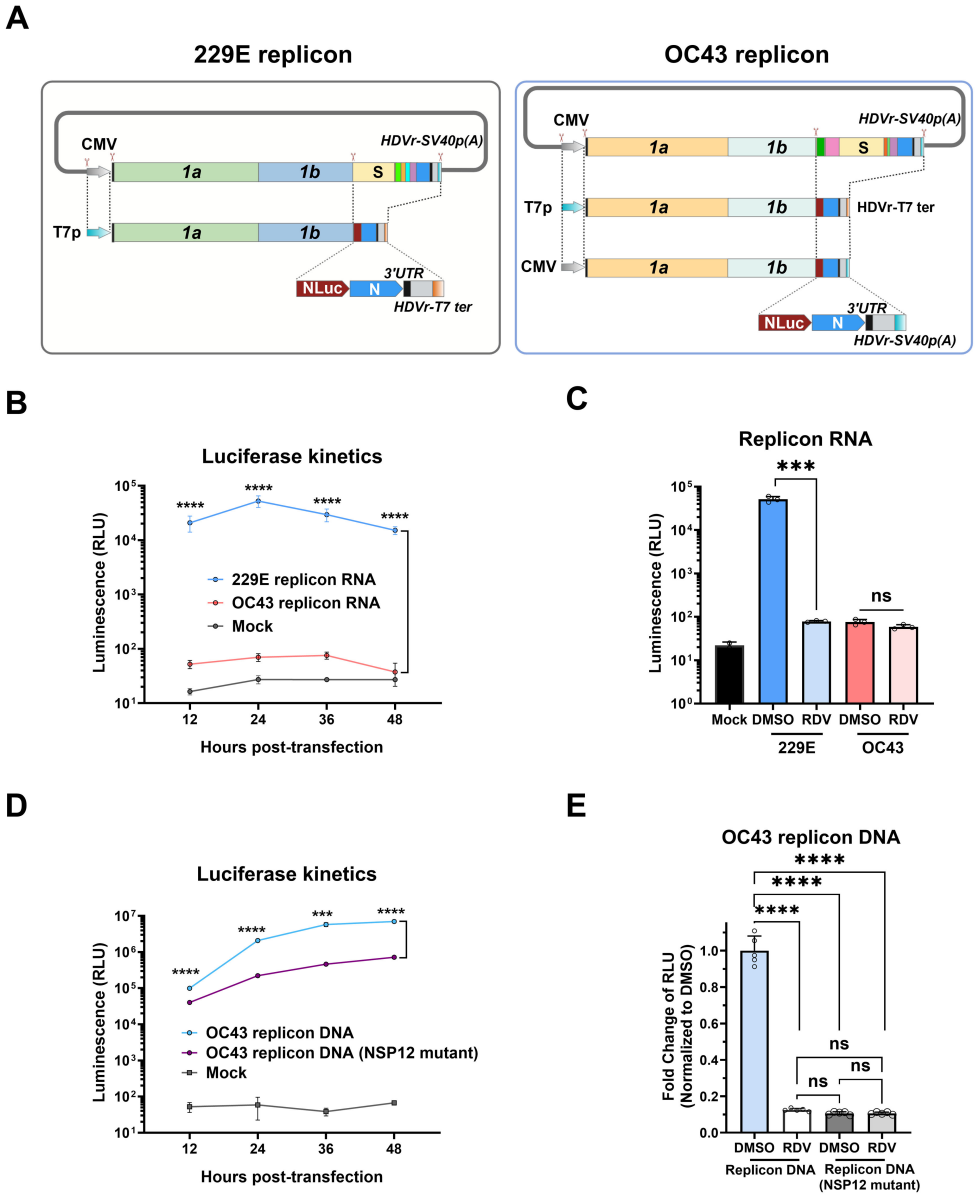
Construction and characterization of 229E and OC43 replicon systems. **(A)** Schematic diagram showing the construction of 229E and OC43 replicon systems. The full-length infectious clones (non-structural, structural, and accessory genes as shown in **Figure 1**) were digested using an *in vitro* CRISPR-Cas9 system to delete the ORFs of structural (S, E, and M) and accessory genes, which were subsequently replaced with the NanoLuc luciferase (NLuc) gene. The replicons were driven by T7 promoter (T7p), and the hepatitis delta virus ribozyme (HDVr) and T7 terminator (T7 ter) were added after the poly **(A)** tail. For OC43, cytomegalovirus (CMV) promoter-driven DNA replicon was also constructed. **(B)** HeLa cells were electroporated with replicon RNAs, and NLuc activity was determined at the indicated timepoints. **(C)** HeLa cells were electroporated with replicon RNAs in the presence of 20 μM remdesivir (RDV) or 0.1% DMSO control, and NLuc activity was measured at 36 h post-transfection. **(D)** HEK-293T cells were transfected with CMV-driven OC43 replicon plasmid, and NLuc activity was measured at indicated timepoints. Mock transfection and OC43 replicon harboring NSP12 mutations (D760N/D761N) were used as controls. **(E)** HEK-293T cells were pre-treated with 20 μM RDV or 0.1% DMSO control for 1 h, followed by transfection with WT OC43 replicon or its NSP12 mutant for 6 **(h)** The medium was then replaced with fresh medium containing 20 μM RDV or 0.1% DMSO for another 18 h for measurement of NLuc activity. *** : P < 0.001, **** : P < 0.0001, ns : not statistically significant.

Replicon systems are valuable tools for studying viral translation and replication, and have been developed for coronaviruses through various strategies (Almazán et al., 2004; Hertzig et al., 2004; Almazán et al., 2006; Ge et al., 2007). Here we constructed replicons for HCoV-OC43 and HCoV-229E to assess compounds that might impact the stage of viral replication. As shown in **Figure 6A**, the workflow for constructing HCoV-229E and HCoV-OC43 replicons is summarized. The replicons included two main open reading frames (ORF1a and ORF1b) and the N gene, which are important for replication, while genes encoding structural (S, E, and M) and accessory proteins were deleted to prevent the generation of infectious viral particles. The NanoLuc luciferase (NLuc) reporter gene was inserted between ORF1b and N, and expressed under the control of transcriptional regulatory sequence (TRS) of the spike gene. The replicons were driven by T7 promoter, with hepatitis delta virus ribozyme (HDVr) and T7 terminator sequences added to the 3’ end of poly (A) to ensure proper transcriptional termination and processing of viral RNA.

Purified replicon RNA from *in vitro* transcription was directly electroporated into HeLa cells, and the NLuc activity expressed from the replicon RNA was measured to evaluate viral replication. Compared to mock-transfected cells, transfection with 229E replicon RNA led to a significant increase in NLuc signal, peaking at 24 h post-transfection and gradually decreased thereafter (**Figure 6B**). In contrast, NLuc signal of OC43 replicon RNA was only slightly above the background of mock-transfected cells. To assess the replicon systems for antivirals, the coronavirus RdRp inhibitor remdesivir was added. Remdesivir significantly reduced the NLuc activity of 229E replicon, but had no impact on the OC43 replicon (**Figure 6C**). These results suggest that RNA-based 229E replicon could be used to assess viral replication, while the OC43 replicon requires further improvement.

To overcome the limitations of OC43 replicon using T7 promoter, an alternative DNA-launched OC43 replicon was constructed by employing the CMV promoter for strong transcriptional initiation (**Figure 6A**). Similarly, the HDVRz and SV40 poly (A) sequences were added to the 3’ end to ensure the production of mature replicon RNA. Due to the continuous transcription activity of the CMV promoter, a replicon containing the mutations of D760N/D761N in NSP12 to inactivate the RdRp was constructed as a control.

The CMV-driven OC43 replicon DNA was transfected into HEK 293T cells. Replicon DNA transfection produced strong expression of Nluc. At 12 h post-transfection, Nluc expression from the OC43 replicon showed a two-fold increase compared to the replicon with NSP12 mutant. At later time points, the difference in Nluc expression was increased by at least 10-fold, indicating that the increased luciferase activity is mainly dependent on viral replication (**Figure 6D**).

Cells transfected with OC43 Replicon DNA were then treated with remdesivir, as described above for replicon RNAs. Interestingly, remdesivir reduced NLuc level in cells transfected with WT replicon DNA by approximately 90% compared to the DMSO control (**Figure 6E**). The Nluc signal was reduced to the level observed in cells transfected with replicon bearing the NSP12 mutant, where Nluc expression is strictly driven by the CMV promoter.

These data demonstrate that T7-driven 229E replicon RNA and CMV-launched OC43 replicon DNA systems provide convenient tools for evaluating antiviral compounds against the coronavirus replication.

## 4 Discussion

In this study, we generated reporter viruses expressing mGreen fluorescent protein for two different mild human coronaviruses, HCoV-OC43 and HCoV-229E, and developed antiviral assays based on high-content imaging analysis. Additionally, we developed replicon systems for each virus using two different strategies and confirmed their feasibility to evaluate viral replication. Reporter viruses developed for coronaviruses, mainly include those expressing fluorescent proteins or luciferases (Shen et al., 2016; Ye et al., 2021). Luciferase expression cassettes inserted into the viral genome are subsequently detected by adding substrates following cell lysis. Here we constructed the fluorescent protein reporter system for non-lytic detection by high-content imaging. We selected the monomeric mGreenLantern, which is known to have the brightest fluorescence among green fluorescent proteins, providing the strong signal under wide-field microscope (Hostettler et al., 2017).

Although the insertion sites for the mGreen reporter gene followed strategy similar to those used in previous work (Cervantes-Barragan et al., 2010; Shen et al., 2016), we employed one-step TAR-cloning in yeast to assemble full-length infectious clones for HCoV-OC43 and HCoV-229E. The rapid assembly of reverse genetics systems for two coronaviruses from different genera demonstrates that TAR cloning can be broadly applied across coronaviruses. The TAR approach enables simultaneous assembly of multiple fragments, offers high efficiency, does not require restriction sites, and ensures sequence stability after assembly-facilitating a rapid response to emerging viral outbreaks.

The expression and genetic stability of the reporter gene were subsequently validated by serial passaging. However, OC43-mGreen reporter virus displayed slightly reduced replication fitness in HRT-18 cells when compared to wild-type OC43, possibly due to compromised ns2a-mediated antagonism of host innate immune responses, as reported in previous studies (Shen et al., 2016).

High-content screening has been widely applied in virological research, including studies on the characterization of the viral life cycle (Banerjee et al., 2013) and phenotypic screening (Moffat et al., 2006; Zhou et al., 2017). By integrating reporter viruses encoding fluorescent protein genes, we then evaluated the dose-response of the well-known coronavirus replication inhibitor remdesivir (Sheahan et al., 2017), calculating the 50% inhibitory concentration (IC_50_). We observed a dose-dependent inhibitory effect of remdesivir on the replication of both reporter viruses, with IC_50_ values in the sub-micromolar range. The results are consistent with previous studies (Parang et al., 2020; Hsu et al., 2021), with improved sensitivity and convenience over cytopathic effect (CPE)-based or other detection methods (Weil et al., 2022).

Furthermore, using the assembled cDNA clones of HCoV-229E and HCoV-OC43, replicon systems were constructed. In terms of replicon construction strategies, transient replicon systems based on *in vitro*-transcribed RNA were considered in this study. Replicon RNA can be introduced into various cell lines to better mimic viral replication under physiological conditions and to evaluate drugs more accurately. Although several stable cell lines harboring the coronavirus replicon RNA have been constructed, cytotoxicity caused by viral proteins during the replication poses challenges for establishing such a system in different cell lines (Hertzig et al., 2004; Liu et al., 2022).

To assess the sensitivity of *in vitro*-transcribed transient replicon systems to remdesivir, an inhibitor of RNA-dependent RNA polymerase, a series of experiments were conducted in HeLa cells. However, only the 229E replicon RNA showed sensitivity to remdesivir treatment. Remdesivir, an adenosine analog, inhibits coronavirus genomic RNA synthesis (Sheahan et al., 2017). At 36 h post-transfection, remdesivir significantly inhibited 229E replicon-dependent Nluc activity, consistent with previous studies (He et al., 2021). As for OC43 replicon, in addition to HeLa cells, we were unable to establish it in HEK-293T and BHK-21 cells (data not shown). One possible explanation is due to the insufficient delivery of full-length OC43 replicon RNA into host cells. Another important factor is that the slow replication of OC43 virus which may require a longer duration of viral replicon RNA in cells to achieve adequate expression of Nluc luciferase for detection. To overcome this, we introduced the CMV promoter and transfected the relatively stable replicon DNA instead of RNA into cells to enable stronger and more sustained transcription of replicon RNA. Through this strategy, increasing activity of Nluc was detected, and the treatment of remdesivir led to approximately a 90% reduction in Nluc activity at 24 h post-transfection. Similar strategy was successfully used to construct the SARS-CoV-2 replicon system (Feng et al., 2022). Although a CMV-based DNA replicon system for OC43 virus has been generated, further optimization is needed to improve the sensitivity of the replicon system.

In summary, we rapidly assembled the full-length infectious clones for two mild human coronaviruses using transformation-associated recombination (TAR) method in yeast, and the recombinant reporter viruses allow for development of high-throughput platform for fundamental research and antiviral screening. Meanwhile, two luciferase-based transient replicon systems were constructed and can be used for the investigations of virus-host interactions or antiviral compounds during coronavirus replication.

## Data Availability

The raw data supporting the conclusions of this article will be made available by the authors, without undue reservation.
